# Access to Sexual Health Services and Support for People with Intellectual and Developmental Disabilities: an Australian Cross-sector Survey

**DOI:** 10.1007/s13178-022-00734-7

**Published:** 2022-06-02

**Authors:** Patsie Frawley, N.J. Wilson, Jennifer David, Amie O’Shea, K. Areskoug Josefsson

**Affiliations:** 1 Faculty of Education, Te Kura Toi Tangata, University of Waikato New Zealand, Gate 1 Knighton Road, Hamilton, New Zealand; 2 School of Nursing and Midwifery, Western Sydney University, Hawkesbury Campus, Locked Bag 3, Richmond, NSW 2753 Australia; 3 Disability & Inclusion, School of Health & Social Development, Deakin University, Gheringhap St, Geelong, 3220 Australia; 4 VID Specialized University, Vågsgaten 40, 4306 Sandnes, Norway; 5 Oslo Metropolitan University, St Olavs Plass, Postboks 4, 0130 Oslo, Norway

**Keywords:** Intellectual and developmental disability, Sexual health, Sexual health training, Sexual health policy, Disability awareness

## Abstract

**Introduction:**

People with intellectual and developmental disabilities under the United Nations Convention on the Rights of Persons with Disabilities (UNCRPD) have the right to access sexual health services including information, education, and support. Little is known about the capacity of sexual health professionals to provide these services.

**Methods:**

Using an observational research design, this study utilised a descriptive survey tool (PASH–Ext) that also encompassed a standardised measure, with a cross-sectional purposive sample of 52 Australian sexual health professionals. Data was collected in 2020.

**Results:**

Just over half of the participants reported having received training in their preservice education to work with people with intellectual and developmental disabilities, of these 60% held the view that people with intellectual and developmental disabilities would not feel embarrassed receiving sexual health information and support.

**Conclusion:**

The study found that training is both important to the professionals’ preparedness to work with people with intellectual and developmental disabilities, and that these professionals advocate for the continuation of this training in pre-service courses and additional training in post service education for sexual health workers.

**Policy Implications:**

To progressively realise Article 25 of the UNCRPD signatory, countries need to ensure sexual health services are accessible to people with intellectual and developmental disabilities. This study recommends that sexual health policy addresses equity of access for people with intellectual and developmental disability by ensuring all staff are prepared and supported to provide these services.

## Introduction

Sexual health is an integral part of living a healthy life (World Health Organization, 2021). Engaging with sexual health as a part of overall health and wellbeing requires an acknowledgement of a person’s sexuality. For people with intellectual and developmental disabilities, this fundamental starting point is not easily achieved. Underpinning this challenge are notions of incapacity and otherness about people with intellectual and developmental disabilities and their sexuality in particular, with narratives of fear dominating their experiences (Frawley & Wilson, 2016). These ideas shape the responses and practices of policy makers, service providers, families, and others who mediate the lives, choices, and decisions of people with intellectual and developmental disabilities. This leads to sex, sexuality, and sexual health of people with intellectual and developmental disabilities being overlooked in service planning, disregarded or minimised in education systems, ignored in policy, and left out of the training of health, sexual health, and other professionals.

The World Health Organization (WHO) has a strong focus on sexual health, promoting equal access to sexual health services for all, and outlining that to achieve sexual health people need:

- access to comprehensive, good-quality information about sex and sexuality;
- knowledge about the risks they may face and their vulnerability to adverse consequences of unprotected sexual activity;
- the ability to access sexual health care;
- to be living in an environment that affirms and promotes sexual health (WHO, 2021 para 1).

People with intellectual and developmental disabilities encounter significant barriers in these areas with particular challenges accessing information in ways they can understand and use, to inform their understanding of sexuality, relationships, and sexual health (Frawley & O’Shea, 2019). Sexuality education is an important conduit to this information. While research suggests there has been an increased interest in sexuality education for people with intellectual and developmental disabilities over the past decade, there are issues with how this education is developed, delivered, and evaluated (Brown et al., 2020). Particular issues are the lack of engagement with people with intellectual and developmental disabilities as educators and a lack of engagement with their lived experiences to guide this education (Alexander & Taylor Gomez, 2017; Brown et al., 2020; Frawley & O'Shea, 2020; McCarthy, 2018; Schaafsma et al., 2017; Stein et al., 2018; Treacy et al., 2018).

Sexuality education for people with intellectual and developmental disabilities has an important role to link people to sexual health services so they can access the expertise of sexual health practitioners. A peer education model in Australia does this by bringing sexual health and sexual assault service professionals into a peer-led programme as ‘program partners’, strengthening connections between people with intellectual and developmental disabilities and mainstream sexuality and sexual health services (Frawley & Bigby, 2014; O’Shea & Frawley, 2019). This approach builds important networks between people with intellectual and developmental disabilities and these services; however, for many people, access to sexuality education programmes is mediated by staff, family, and others who may not always prioritise sexuality and sexual health (Chrastina & Večeřová, 2020).

Researchers have explored the living, learning, and support environments that surround people with intellectual and developmental disabilities and the importance of these environments in affirming and promoting sexuality rights and enabling access to information and support for sexual health (Björnsdóttir & Stefánsdóttir, 2020; Brown & McCann, 2019; Kammes et al., 2020; Maguire et al., 2019; Muswera & Kasiram, 2019; Neuman, 2020; Wilson & Frawley, 2016). This work indicates that while there has been some progress towards supporting the sexuality and sexual health of people with intellecutal and developmental disabilities in these environemnts, the main barrier to having sexuality acknowledged and supported for people with intellectual and developmental disabilities remains the attitudes of ‘those around them’ in these environments. Primarily, it is the views of support staff in these environments that hold that people with intellectual disabilities do not have ‘the capacity’, for ‘self determined’ or what they see as ‘appropriate’ sexual expression that leads to practices that subsequently restrict sexual expression. Michael Gill (2015) refers to this as ‘sexual abelism’ which is based on a view that the sexuality of people with intellectual and developmental disabilities is ‘not like ours’ (where ‘ours’ refers to people who are not identified as having an intellectual or developmental disability). The thesis of his book *Already Doing it* is that restrictive environments, ‘…might deny recognition of sexual citizenship’ (p.8), but people with intellectual and developmental disabilities, like most people, are sexual (including those who seek intimacy rather than sexual activity) and have sexual agency. He notes ‘Sexual pleasure can emerge in spaces where sexual ableism operates’ (p.6), highlighting that the formal restriction or policing of sexual expression of people with intellectual and developmental disabilities will not ‘stop’ people being sexual and therefore needing access to sexual health information and services.

Supporting this view, Carter and colleagues in their 2021 review of two decades of the research literature about sexuality experiences of people with intellectual disabilities reported that when people with intellectual and developmental disabilities were asked about their sexuality they described the same range and scope of sexuality desires, interests, and identities as other people without this label (Carter et al., 2021). However, respondents in this research also reported additional experiences relating to social isolation, lack of access to education and information, and restrictions on sexual expression. This research describes a mismatch between the aspirations held by people with intellectual disabilities for a full and healthy sexual life, and the way they are perceived and responded to as incapable of choosing or determining their sexual lives at best, and more commonly not being acknowledged as sexual at all. This has significant implications for how sexual health of people with intellectual and developmental disabilities is considered by sexual health policy and how people are responded to in sexual health provision.

Research suggests that this lack of attention to the sexuality and sexual health of people with intellectual and developmental disabilities is leading to negative sexual, reproductive, and sexual health outcomes for them. These include increased risk of sexually transmitted infections (Schmidt et al., 2019), forced contraception and sterilisation (Elliott, 2017), and unplanned pregnancy and limited parenting support (Lightfoot & DeZaler, 2020). Furthermore, for LGBTQIA + people with intellecutal and developmental disabilities, there are additional complexities in having their sexualities acknowledged as a precursor to accessing sexual health information and services (Robinson et al., 2020).

Access to sexual health services, including services that provide relationship education, counselling, and sexual assault support, needs to be addressed to ensure people with intellectual and developmental disabilities have an equal opportunity for sexual health–supporting services (Schmidt et al., 2021). This right is enshrined in the United Nations Convention on the Rights of Persons, Article 25, which states that States Parties, ‘ Provide persons with disabilities with the same range, quality and standard of free or affordable health care and programmes as provided to other persons, including in the area of sexual and reproductive health…’ (UN, 2007). This requires signatory countries like Australia to progressively realise this right in policy and practice.

There is limited research about the capacity and preparedness of health practitioners to provide sexual health services to people with intellectual and developmental disabilities. A recent systematic review by Pelleboer-Gunnink et al. (2017) reported that mainstream health practitioners held stigmatising attitudes towards people with intellectual disabilities, attitudes which then impacted the delivery of services. In particular, health practitioners failed to recognise and then respond to the ways in which people with intellectual disabilities might require adaptations to their established practice. The authors report a lack of health professionals’ knowledge of intellectual disabilities, due primarily to limited experience with people so-labelled and a lack of training and education about, and informed, by the lived experiences of people with intellectual disabilities. Without experience or training, health professionals are more likely to be informed by their attitudes to sexuality and intellectual disability.

Research over a number of decades has sought to understand the attitudes of staff, families, and the general public towards the sexuality of people with intellecutal and developmental disabilities. Gill (2015) notes this is anaolgous to research over the same period having failed to seek the views of people with intellectual disabilities themselves. What that resarch has confirmed though is that generally the views held by ‘others’ towards the sexuality of people with intellecutal disabilities is negative, protective, and unsupportive of their sexual citiezenship (Gill, 2015; Deffew et al., 2021). Recent research with healthcare providers in the USA found that while healthcare providers aimed to give sexual health and relationship information, education, and services to people with intellecutal and developmental disabilities they faced four key barriers: ‘(1) clients’ level of understanding, (2) providers’ lack of knowledge about or access to appropriate resources, and (3) providers’ lack of knowledge about or access to appropriate referrals’ (Schmidt et al., 2021, p. 5). Furthermore, this research found that where healthcare providers did provide sexual and reproductive health, many provided it to the persons’ parents rather than directly to people with intellectual and developmental disabilities themselves (p.5).

In most parts of Australia, specialist sexual health services are provided by Family Planning Associations which are members of the Family Planning Alliance Australia and the International Planned Parenthood Federation. These services do offer some disability-specific information, support, and education in the key areas of their work including contraception, women’s health screening, and sexually transmitted infection (STI) education; however, there are no specialist sexual health services for people with disabilities. Many people with disabiltieis like other Australian citizens also access sexual health services through their General Practioner or community health provider. There is very limited research in an Australian context about sexual health service provision to people with intellectual and developmental disabilities; however, research with young people with physical disabilities found that health professionals lacked confidence, knowledge, and skills, or perceived they did not have what they understood to be the ‘specialist’ knowledge and skills to provide services to young people with physical disabilities (Ride & Newton, 2018).

## Aims

The research reported in this paper aimed to:

- Describe the self-reported confidence and skills of a sample of Australian sexual health and/or sexual assault professionals in providing information and services about sexual health and sexual issues to people with intellectual and developmental disabilities;
- Identify if these professionals require more training and/or experience to feel comfortable and confident providing sexual health services to people with intellectual and developmental disabilities.

## Method

### Survey Design

Using an observational research design, this study utilised a descriptive survey tool that also encompassed a standardised measure, with a cross-sectional purposive sample of Australian sexual health and sexual assault professionals.

### Survey Tool

The survey tool was primarily based on *the Student’s Attitudes towards addressing Sexual Health* (SA-SH) (Areskoug-Josefsson et al., 2016). The SA-SH has been further developed to an extended version SA-SH-Ext (Lunde et al., 2020) and to a version for professionals, *Professionals’ Attitudes towards addressing Sexual Health* (Elnegaard et al., 2020) (PASH). The extended version is composed of 27 items where 22 are from the original SA-SH. The SA-SH addresses allied health and health students’ attitudes towards addressing sexual health issues and the PA-SH survey focuses on professionals in their current professional role. The 27 items are distributed over 4 domains: Present feelings of comfortableness (items 1–13), Working environment (items 14–19), Fear of negative influence on patient relations (item 20–22), and Educational needs (items 23–27).

Items are responded to on a Likert-type scale with five response options: disagree, partly disagree, partly agree, agree, and strongly agree.

The SA-SH has shown good validity and reliability in previous studies (Areskoug-Josefsson et al., 2016, 2019a, b, 2018; Gerbild et al., 2017; Turan et al., 2021). The SA-SH-Ext has only been pilot-tested showing similar validity as the SA-SH (Lunde et al., 2020). The SA-SH has been used to evaluate educational interventions, such as short courses in Sexual Health for students (Felter, 2020; Gerbild et al., 2018).

The PASH-Ext was modified by the Australian authors (PF, AO, NW) to ensure that the questions were specific to the Australian context, replacing terminology such as ‘patients’ with ‘clients’, using person-first language, and adopting the term ‘intellectual and developmental disability IDD’ throughout. Australian-specific demographic items were also added to the survey tool. The survey tool consisted of demographic items (age, gender, profession, sector, years’ experience, postcode, and highest qualification) and IDD-specific education items (did undergraduate training have IDD-specific content and, if so, did this include sexual health?).

### Ethical Considerations

The study was approved by Deakin University Low Risk Human Research Ethics Committee (Approval ID: HEAG-H 93–2020).

### Participants

The inclusion criteria for this study were that participants were professionals who worked in Australian sexual health, sexual assault, women’s health, and other related health or social services where the research team knew of these services from previous research, and/or had knowledge of them in their local areas. These included, for example a specialist violence and abuse service for women with disabilities, a private disability and sexual health educator, and a disability support service that offered sexuality and relationships education. There was no threshold for years of experience or professional qualification.

### Procedure

A purposive sampling approach was used to collect all data. The survey data were collected using Qualtrics© between July 2020 and September 2020. An anonymous survey link was distributed via email to 111 services that employed staff who met the inclusion criteria. This list was collated after undertaking an online search of publicly listed government and private health services in each State and Territory of Australia with the search terms sexual health, sexual assault, women’s health; this list was extended with input from Australian members of the research team (PF, AO, NW, MW) who had knowledge of services through previous research and/or knowledge of services in their local areas After 3 weeks of the survey being open, a series of follow-up phone calls were made by Australian members of the research team (PF AO, NW, MW), to 36 listed services across Australia in the state/region of the researcher, in order to spread the word more widely about the survey to increase participant numbers. It is difficult to estimate the whole cohort size of professionals across Australia that were potential participants as each state and territory has totally different health and social service systems and the composition of teams are not publicly available. In the research planning, it was estimated that the 111 sites identified was a fair representation of the sites that met selection criteria. We were not able to establish how many staff at each of these sites would have met the inclusion criteria.

### Data Analysis

IBMM SPSS Statistics (Version 26) was utilised for all descriptive and inferential data analysis of the survey data. The data were initially cleaned and, if required, string variables were re-coded and/or recategorised. All five-item Likert responses to the PASH-Ext were recategorised into three items, where strongly disagree and disagree were collapsed into a single ‘negative’ item, partly agree was re-labelled as ‘neutral’, and agree and strongly agree were collapsed into a single ‘positive’ item. The original SA-SH (Areskoug-Josefsson et al., 2016) used two categories, including the neutral option as positive, but this was changed after a Rasch-analysis of the SA-SH (Areskoug-Josefsson & Rolander, 2020).

## Results

A total of 64 survey responses were received, including 12 which were incomplete and removed from any analysis. This left 52 complete responses consisting of a wide range of health and social professionals; 57% noting they worked in the health sector including 16.3% in Sexual Health while 21% reported they worked in sexual assault and family violence sector with the remainder working in Allied Health, Education, and other non-identified sectors. Many respondents had a post-graduate qualification (51.9%) with a reported median number of 13 years’ experience in the sector. The largest proportion of respondents (*n* = 15; 28.8%) were aged between 35 and 45 years, with the youngest participant (*n* = 1; 1.9%) in the 18–25 years category, and the eldest participants (*n* = 13; 25%) aged 55 years or over. As expected, most responses came from populous Australian states on the eastern seaboard. Of note was that just under half of all respondents (*n* = 24; 46.2%) reported having some education about intellectual and developmental disabilities in their undergraduate courses and, of these, almost half (*n* = 11; 45.8%) reported also having sexual health and intellectual and developmental disabilities–specific content. Table 1 lists all of the demographic data.

**Table 1:** Demographic data (*N* = 52)

**Category**	**Frequency**	**Percentage (%)**
**Gender** * Male* * Female* * Non-binary*	4 47 1	7.7 90.4 1.9
**Profession** * Counsellor* * Advocate* * Educator* * Health promotion officer* * Nurse* * OT* * Doctor* * Other*	14 1 5 9 10 5 2 6	26.9 1.9 9.6 17.3 19.2 9.6 3.8 11.5
**Sector (multiple entries)** * Education* * Health* * Allied health* * Sexual assault service* * Sexual health service* * Government* * Counselling* * Advocacy* * LGBTIQA* + *service* * Support coordination* * Disability support service* * Family violence service* * Other*	9 29 9 13 17 2 8 8 3 1 1 4 7	17.3 55.8 17.3 25 32.7 3.8 15.4 15.4 5.8 1.9 1.9 7.7 13.5
**Years of experience** * 0–5* * 6–10* * 11–15* * 16–20* * 21–25* * 26–30* * 31* +	12 11 8 6 6 7 2	23.1 21.2 15.4 11.5 11.5 13.5 3.8
**Intellectual disability training** * Yes* * N0*	24 28	46.2 53.8
**Sexuality and disability training (*N* = 24)** * Yes* * No*	11 13	45.8 54.2
**Location** * NSW/ACT* * VIC* * QLD* * WA* * SA* * TAS* * NT*	16 18 11 2 3 1 1	30.8 34.6 21.2 3.8 5.8 1.9 1.9

### PASH-Ext (Australian Version)

All responses to the PASH-Ext items are listed in Table 2. For the PASH-Ext items relating to attitudes towards clinical practice, responses were primarily as expected. That is, comfort levels for all of the items asking about comfort (items 1–12) were high. Items 15 and 16–both about how people with intellectual and developmental disabilities might feel in relation to sexual health–were the only items where attitude responses were more varied, indicating that a greater proportion of professionals were less certain that people with intellectual and developmental disabilities might not feel embarrassed/uneasy about sexual health issues. Responses to items 25 and 26 indicate that professionals feel both competent and confident in working with people with intellectual and developmental disabilities in relation to sexual health. Nevertheless, 40% of respondents felt that they did not receive enough basic training about sexual health and 38.5% reported wanting more education about sexual health.

**Table 2:** PASH item, responses (*N* = 52)

**PASH item**	**Negative**	**Neutral**	**Positive**
1. I feel comfortable informing clients about sexual health	0	1 (1.9%)	51 (98.1%)
2. I feel comfortable initiating a conversation regarding sexual health	0	3 (5.8%)	49 (94.2%)
3. I feel comfortable discussing sexual health with any of my clients	1 (1.9%)	5 (9.6%)	46 (88.5%)
4. I feel comfortable discussing sexual health with clients with multiple disabilities, including physical disability	0	6 (11.5%)	42 (80.8%)
5. I feel comfortable discussing sexual health with clients with physical illness	0	6 (11.5%)	46 (88.5%)
6. I feel comfortable discussing sexual health with clients with intellectual/developmental disability	3 (5.8%)	6 (11.5%)	43 (82.7%)
7. I feel comfortable discussing sexual health with clients with mental illness	2 (3.8%)	5 (9.6%)	45 (86.5%)
8. I feel comfortable discussing sexual health issues with clients regardless of their gender identity	0	4 (7.7%)	48 (92.3%)
9. I feel comfortable discussing sexual health issues with clients with ID & DD regardless of their age	3 (5.8%)	7 (13.5%)	42 (80.8%)
10. I feel comfortable discussing sexual health issues with clients regardless of their cultural background	4 (7.7%)	6 (11.5%)	42 (80.8%)
11. I feel comfortable discussing sexual health issues with clients with ID & DD regardless of their sexual orientation	2 (3.8%)	5 (9.6%)	45 (86.5%)
12. I feel comfortable discussing specific sexual activities with clients with ID & DD	3 (5.8%)	14 (26.9%)	35 (67.3%)
13. I am unprepared to talk about sexual health with clients with ID & DD	37 (71.2%)	6 (11.5%)	9 (17.3%)
14. I believe that I might feel embarrassed if clients with ID & DD talk about sexual issues	45 (86.5%)	5 (9.6%)	2 (3.8%)
15. I believe that clients with ID & DD might feel embarrassed if I bring up sexual issues	21 (40.4%)	22 (42.3%)	9 (17.3%)
16. I am afraid that clients with ID & DD might feel uneasy if I talk about sexual issues	29 (55.8%)	15 (28.8%)	8 (15.4%)
17. I am afraid that conversations regarding sexual health might create a distance between me and my clients with ID & DD	47 (90.4%)	4 (7.7%)	1 (1.9%)
18. I believe I have too much to do in my role to have time to handle sexual issues	49 (94.2%)	2 (3.8%)	1 (1.9%)
19. I take time to deal with clients with ID & DD’s sexual issues in my professional role	4 (7.7%)	11 (21.2%)	37 (71.2%)
20. I am afraid that my colleagues would feel uneasy if I brought up sexual issues with clients with ID & DD	44 (84.6%)	5 (9.6%)	3 (5.8%)
21. I am afraid that my colleagues would feel uncomfortable dealing with questions regarding clients with ID & DD sexual health	40 (76.9%)	6 (11.5%)	6 (11.5%)
22. I believe that my colleagues will be reluctant to talk about sexual issues for clients with ID & DD	38 (73.1%)	6 (11.5%)	8 (15.4%)
23. Sexual health was a part of my professional training	17 (32.7%)	4 (7.7%)	31 (59.6%)
24. I believe that I received basic knowledge about sexual health in my training/education	21 (40.4%)	5 (9.6%)	26 (50.0%)
25. I have sufficient competence to talk about sexual health with my client with ID & DD	4 (7.7%)	8 (15.4%)	40 (76.9%)
26. I believe in my own ability to promote sexual health in clients with ID & DD in my professional role	5 (9.6%)	6 (11.5%)	41 (78.8%)
27. I think that I need to be trained in my education to talk about sexual health	22 (42.3%)	10 (19.2%)	20 (38.5%)

In order to explore if any PASH-Ext items were dependant on a number of independent variables (IVs), chi-square tests were run using the following IVs against all PASH-Ext items: age, gender, profession, sector, years’ experience, and whether respondents had received any intellectual and developmental disability–specific training in their undergraduate degree. As the sample size was small, almost all chi-square tests failed the minimum assumptions for applying the statistical test. Nevertheless, for specific training in the undergraduate degree IV, the assumptions of the chi-square test were met for PASH-Ext items 6, 12, 13, 15, 16, 19, 20, 21, 22, 23, 24, 26, and 27. For all of these items, except for item 15 (*I believe that clients with ID & DD might feel embarrassed if I bring up sexual issues*), the chi-square test of dependence showed that responses to PASH-Ext items were not dependent upon whether respondents has received intellectual and developmental disability–specific training in their undergraduate degree. For item 15, respondents who had received such training were more likely to report (66.7%) that people with IDD would not feel embarrassed, and this relationship was significant: *Fisher’s exact test* = 6.66, *p* = 0.034, *V* = 0.36.

## Discussion

This exploratory study about the self-reported level of preparedness, confidence, and capacity of sexual health– and sexuality-related professionals to provide services to people with intellectual and developmental disability provides new information that can be used to inform future research in this area. As outlined in the background to this paper, people with intellectual and developmental disabilities, when they are asked, share their aspirations and experiences of being sexual like everyone else. Barriers to a healthy sexual life exist primarily in the attitudes held by others about sexuality in the lives of people with intellectual and developmental disabilities that lead to restrictions to sexual expression, and the view that people with intellectual and developmental disabilities do not have sexual agency. The ‘extraordinary sexuality’ that Gill (2015) refers to is based on a view that people with intellectual and developmental disabilities are not self-determined or agentic in relation to their sexuality, but inherently different in a way that contributes to their invisibility within sexual health and support services.

Research referred to earlier suggests that knowing about intellectual and developmental disability either professionally or personally (Schmidt et al., 2019) is a likely benefit for health professionals feeling prepared to work with people with intellectual and developmental disabilities. The PASH-Ext (Australian version) survey provided an opportunity to find out if preparedness of sexual health and sexual health–related professionals in Australia was one of the barriers resulting in sexual health inequity.

A noted finding of the PASH-Ext (Australian version) survey was that just under half of the professionals surveyed had received training in their undergraduate degrees relating to people with intellectual and developmental disabilities and of these almost half had received content specifically related to sexual health and intellectual and developmental disability. While it is possible that those with this experience felt more confident responding to the survey, this theme is interesting and an important one to highlight. These professionals may have been able to draw on the knowledge gained from their undergraduate training to underpin their practice with people with intellectual and developmental disabilities. Additionally, the statistical testing was significant in relation to their view that people with intellectual and developmental disabilities would not feel embarrassed when receiving sexual health information and other services from them. Over 60% of those who had received specific training about intellectual and developmental disabilities held this view. Despite this level of confidence and preparedness to provide services to people with intellectual and developmental disabilities, the need for more training about sexual health was reported by 40% of respondents. This suggests information about sexuality and sexual health relating to people with intellectual and developmental disabilities needs to continue to be included in medicine, nursing, and allied health undergraduate and other training programmes, be developed in those that do not cover this information, and be embedded in professional development for this group of health professionals.

It is interesting to note that most of the respondents for the study were from the Australian eastern seaboard and were predominantly female (90.4%). These two factors are most likely characteristics of sexual health service provision in Australia; however, this study did not interrogate these factors so we cannot provide any further reflection on the effect of them on the study and its findings. Furthermore, as noted in the results section, we are drawing on a small sample of a small cohort of service providers in Australia. It is not possible to identify the total number of sexual health providers in Australia who met our inclusion criteria; however, this small sample in this exploratory study does indicate that training about sexual health and intellectual and developmental disabilities in undergraduate health– and health-related programmes is beneficial to future practitioners. This is an important finding that may inform future research and programme development in these programmes.

## Limitations

There are a number of limitations to this study; the main one was a low response rate, which may indicate that professionals, who did not consider the topic important or felt less comfortable in addressing sexual health issues, did not respond to the survey. Strategies to increase the response rate in future research of this nature could include more in-depth contact with services, such as by attending staff meetings, where the rationale for the study could be more clearly articulated rather than the likelihood that many potential participants might delete an email from an unknown source. Nevertheless, such strategies are cost- and labour-intensive which precludes their use in small unfunded studies across a large continent like Australia.

Although we interpret the results with caution, this remains the first Australian survey of mainstream health professionals and offers some noteworthy, if not generalisable issues as a base for further research. The survey was sent out during the COVID-19 pandemic, which may have affected the response rate.

## Conclusion

The United Nations Convention on the Rights of Persons with Disabilities codifies the right of people with intellectual and developmental disabilities to access sexual health services equitably with others in their community. Previous research has shown that professionals often lack those competencies, and that persons with intellectual and developmental disabilities often lack sufficient support from professionals concerning sexual health. However, this research, while exploratory suggests a high level of preparedness and sense of capability in the health professionals surveyed. There is a need to extend this research to include larger numbers of participants, combined with qualitative research methods to learn more from professionals about their practice. In addition, qualitative research with people with intellectual and developmental disabilities about their experiences of sexual health is needed to inform training and to ensure optimised support of sexual health for persons with intellectual and developmental disabilities.

## Data Availability

Not applicable.
